# Construction and application on the training course of information literacy for clinical nurses

**DOI:** 10.1186/s12909-023-04505-9

**Published:** 2023-08-29

**Authors:** Chao Wu, Yinjuan Zhang, Jing Wu, Linyuan Zhang, Juan Du, Lu Li, Nana Chen, Liping Zhu, Sheng Zhao, Hongjuan Lang

**Affiliations:** 1 Department of Nursing, Air Force Medical University, No.169 Changle West Road, Xi’an, 710032 Shaanxi China; 2 Department of Anesthesia Intensive Care Unit, The Second Affiliated Hospital, Air Force Military Medical University, Xi′an, Shaanxi China; 3 Department of Otolaryngology, Army Hospital of the Seventy- seventh Group, Jiajiang, Sichuan China; 4 Department of Foreign Languages, Quzhou NO. 2 School, Quzhou, Zhejiang China; 5 Department of Political Teaching, Quzhou NO. 2 School, Quzhou, Zhejiang China

**Keywords:** Clinical nurses, Information literacy, Training courses, Delphi, Empirical research

## Abstract

**Design:**

A two-round Delphi survey was conducted to seek opinions from experts about the index system for the evaluation of training courses of clinical nursing staff’s information literacy. Besides, a non-randomized controlled experimental study was adopted to check the application effect of the training courses.

**Aims:**

This study intended to construct a training course of information literacy for clinical nurses, train nurses in order to improve their information literacy level and provide theoretical reference for the training of information literacy courses for clinical nurses.

**Methods:**

Two rounds of Delphi study were conducted for the study among 26 clinical medical and nursing experts as well as educational experts from 5 different provinces and cities in China. From July 2022 to October 2022, a total of 84 clinical nurses from two hospitals were selected by the convenience sampling method, of which the nurses in one hospital were the control group and the nurses in the other hospital were the observation group. 42 nurses in the observation group were trained by the constructed information literacy training course. Questionnaire evaluation was used to compare the differences in the level of information literacy of nurses and the training effect between the two groups.

**Results:**

The results of the Delphi consultation showed that the expert’s judgment coefficient was 0.958, the expert’s familiarity was 0.946, and the expert’s authority coefficient was 0.952. Finally, a training course of information literacy for clinical nurses with 4 course categories and 45 specific course contents was formed. Among them, nursing information awareness included 7 courses, nursing information knowledge 15 courses, nursing information ability 19 courses, and nursing information ethics 4 courses. The results of the empirical study showed that the information literacy level of the nurses in the observation group after the training of the information literacy course was improved, and the scores in nursing information awareness, nursing information knowledge, nursing information ability, and information ethics were significantly higher than those in the control group after training (*P* < 0.05).

**Conclusions:**

The constructed information literacy training courses for clinical nurses were clearly targeted and systematic. Empirical research showed that the course contents were scientific and reasonable, which could provide reference for the training of clinical nurses’ information literacy.

## Introduction

With the rapid development of information technology, massive amounts of information are generated, circulated, exchanged, and exported at every moment [1, 2]. In clinical work, nurses have to face and deal with massive amounts of information every day [3]. Evidence-based practice is a set of basic practical frameworks in which clinical nursing work practices are based on the outcomes of scientific research and best practices emerge from the available evidence, which is important in clinical work. Nurses are more likely to need information literacy in the context of Evidence-Based Practice which is changing the way of work and thinking patterns of nurses, and put forward new requirements for the ability of clinical nurses to collect, process and utilize information.

Information literacy, being a concept, was first posed in 1974 by Paul Zurkowski, Chairman of AIIC (American Information Industry Committee). The essence of information literacy is a basic ability required by people in the global information age which refers to the ability to perceive, obtain, evaluate, and use information creatively [4, 5]. Good information ability can help nurses improve the efficiency and quality of clinical nursing work. At the same time, it can help nurses better deal with problems in clinical nursing work [6]. Nowadays, the use of information systems has become a central part of a nurse’s work and research showed that nurses’ high documentation and information competence were associated with fewer detected information-related errors [7]. In order to meet the challenges in the era of big data, it is urgent to improve the information literacy of nursing staff [8].

Research showed that most Chinese nursing university students had a low level of information literacy, lacked keen information awareness, and were not good at using scientific and technological achievements, so they could not keep abreast of the latest research results in this professional field in time [9]. Clinical nurses are not good at fully and correctly expressing their own information needs due to lack of information literacy. In clinical nursing work, the purpose of nurses to obtain information is not very clear, and most of them lack specific and in-depth information needs [10]. At the same time, most of them are not good at literature retrieval and lack of nursing research ability [11]. In nursing research, they mainly focus on Chinese literatures, and are lack of the ability to retrieve foreign language literature information of nursing [12]. Hence the information literacy of Chinese clinical nurses is not optimistic and need further training and improvement. It is urgent to developing nurse education in the field of information literacy.

However, the current textbooks in China focus on the cultivation of nurses’ clinical nursing ability and the teaching of medical knowledge [13, 14]. In the education of nurses in China, there were relatively few specialized teaching materials on information education of nurses, focusing more on other aspects of nurse education [15]. The information literacy education of nurses often relied on the textbooks of medical information education, which was lack of professionalism of nursing information education [16]. Besides college education, further continuing education on nursing in hospitals normally did not include such information literacy courses systematically [17, 18]. Insufficient attention was paid to the skills training of nurses’ information literacy, especially information awareness and application of information resources [19, 20]. To our knowledge there was no relevant research on specialized information literacy training courses for nursing staff, and there was no standardized training model for reference.

Therefore, the purpose of our study was to construct a training course and develop nurse education in the field of information literacy, and conduct empirical research to improve their information literacy level so as to provide theoretical reference for the training of information literacy courses for clinical nurses.

## Methods

### Construction of information literacy training courses for clinical nurses

#### Setting up research group

The group was composed of 6 personnel, including 2 professors, 2 graduate students and 2 clinical head nurses. Research group searched the literature on nurses’ information literacy, and conducted qualitative interviews with nursing experts and education experts to clarify how many aspects of information literacy nurses should have, and held a group meeting to determine the first draft of the training course of information literacy for clinical nurses. This group also compiled the consultation questionnaire, selected experts, distributed and collected the questionnaire and analyzed the experts’ opinions.

#### Constructing consultation questionnaire

Delphi expert consultation questionnaire consisted of 3 parts: (a) basic information of the experts, including age, work unit, year of working, educational background, titles and so on; (b) training courses of information literacy for clinical nurses. The first draft was formed on the basis of the search of information literacy related literature. Then we interviewed nursing and education experts, summarized the interview results, and finally formed the first draft through group discussion. The first draft constructed contained 4 courses categories and 33 courses contents. The importance of each level was assigned by the Likert 5-level scoring method, and “point 5 to 1” corresponded to “very important to very unimportant”; (c) expert familiarity with content of the survey and judgement.

#### Selection of the experts

The number of consultation experts was normally between 15 and 50 [21]. And the more experts there was, the more reliable the result would be. The inclusion criterion of the experts in this study were: (a) specialists in clinical medicine and nursing: they should be engaged in clinical medicine or nursing for more than 15 years, obtain the intermedium or advanced level of certificate, acquire a bachelor degree or above, sign the informed consent form and voluntarily participate in this study; (b) specialists in nursing education: they should be engaged in nursing education for more than 15 years, obtain the intermedium or advanced level of certificate, acquire a bachelor degree or above, sign the informed consent form and voluntarily participate in this study.

#### Conducting expert consultation

On August 2020, the research group launched the first round of Delphi consultation to the selected experts. The consultation questionnaire was sent via E-mail to the experts. After the first round of questionnaires were collected, the index items were analyzed with reference to expert opinions. The index items were analyzed according to the criteria that the coefficient of variation should be less than 0.25 and the mean should be greater than 3.5 [22]. If the coefficient of variation is large, it indicates that there is a disagreement among experts on the item. Based on the first round of consultation, items were deleted, modified and added to form the second round of consultation questionnaire. On September 2020, the second round of consultation questionnaire was carried out. The experts were also given two weeks to fill in the questionnaires.

### Implementing the training courses of information literacy for clinical nursing staff

#### Participants

In this research, a non-randomized controlled experimental study design was adopted and the required sample size was calculated by software G*Power 2.1. Repeated measures analysis of variance statistic method was chosen to estimate the sample size. According to the research purpose and design, the effect size was 0.8, α was 0.05, power was 0.95, allocation ration was 1 and the total sample size was 84.

From July 2022 to October 2022, a total of 84 clinical nurses from two hospitals were selected by convenience sampling method, the selected nurses in one hospital was the control group while the selected in the other hospital was the observation group, with 42 in each. The inclusion criteria for the clinical nurses were: having nursing qualifications, being engaged in clinical nursing work, signing informed consent form and voluntarily participating in the study. The exclusion criteria were: those who were nursing staff for internships, who were not on duty due to illness, personal affairs, maternity leave or business trips, and who may go out to study during the research period.

#### Research implementation

The two selected hospitals were two affiliated hospitals of the same university so the content of continuing education in both hospitals were similar. In the empirical study, the control group did not carry out systematic information literacy training, and they only participated in the relevant continuing education, while the observation group, apart from the regular continuing education and training, was trained as per the courses of information literacy for nursing staff constructed in the previous stage. Theoretical knowledge was mainly trained by classroom lectures and group discussions; information retrieval was trained by demonstration and computer operation; evidence-based nursing knowledge was trained by document retrieval and document quality evaluation.

##### Qualifications for lecturers

In order to ensure the quality of training, lecturers needed to have the following qualifications: obtaining a bachelor degree or above, having intermediate or above professional titles. When the course contents were settled, qualified lecturers were selected and provided with half a year to prepare for the lectures before they carried out the training.

##### Teaching methods

At present, public gradually concern the effectiveness of classroom instruction. The more diversified and rationalization of the teaching models and teaching methods we adopt, the more positivity and creativity of nurses could be stimulated. So, the teaching of information literacy for nurses was not limited to the traditional face-to-face teaching method. Diversified forms of teaching methods were adopted, such as group discussion and reports, lectures, flipped class and computer-based operations.

##### Evaluation methodology

Nurses’ information literacy is measured by self-report questionnaire measurement which is a questionnaire of confidence. The information literacy scale (Chinese version) was translated and adapted by researchers through expert meetings and discussions based on Wadson and Cantwell’s researches [4, 23]. The scale has good reliability and validity among Chinese people [24, 25], and has been validated in our previous study [26]. The information literacy scale of nurses was used to investigate the two groups of nurses before and after the training. The scale contained 5 dimensions and 30 items: information awareness (8 items), information knowledge (6 items), information ability (4 items), information ethics (6 items) and information support (6 items). The 5-level scoring method which means a Likert scale set of questions was adopted. From “fully consistent with the item” to " completely disagree with the item “, the scale was scored from “5 point” to “1 points”. The higher the score of the questionnaire, the higher the nurses’ information literacy.

In July and October 2022, that is, before the start of the study and after the implementation of the training course, we distributed paper questionnaires to the two groups of nurses to investigate their information literacy. After the investigation, the questionnaires were checked one by one, and the invalid questionnaires, that is, the selected answers were identical or the answers were inconsistent, were excluded. The differences in the information literacy of the two groups of nurses before and after training were compared.

## Results

### Construction on the training course of information literacy for clinical nurses

#### General information of the experts

26 specialists in clinical medicine and nursing, and nursing education from 5 provinces and cities including Shaanxi, Beijing, Shanghai, Chongqing and Zhejiang were selected. The age of the experts involved ranged from 36 to 57 years old, with an average of 45.92 (SD 4.98) years old. Their working years ranged from 15 to 33 years, with an average of 21.38 (SD 4.91) years. The details were shown in Table 1.

**Table 1:** Demographic information of experts(N = 26)

Item		Frequency(N)	Proportion(%)
Age (years)	<40	3	11.54
	40 ~ 50	18	69.23
	>50	5	19.23
Working experience (years)	<20	7	26.92
	20 ~ 30	17	65.38
	>30	2	7.69
Educational background	Doctorate	8	30.77
	Master	13	50.00
	Bachelor	5	19.23
Professional title	Senior level	9	34.62
	Associate-senior level	13	50.00
	Intermediate level	4	15.38
Research field	Clinical medicine and nursing	17	65.38
	Nursing education	9	34.62

#### Experts’ enthusiasm

The enthusiasm of the experts was assessed by way of the response rate of the questionnaires. In the first round of expert consultation, 28 questionnaires were distributed while 26 valid questionnaires were returned, with a response rate of 92.86%. In the second round, 26 questionnaires were distributed while 26 valid questionnaires were returned, with a response rate of 100%. In the two rounds of consultation, 16 and 3 experts respectively put forward their suggestions on the revision of the course items, indicating that the experts had a good enthusiasm in this study. The details were shown in Table 2.

**Table 2:** Response rate of the questionnaire

Field of the expert	First round				Second round		
	Number of questionnaires distributed	Number of responses	Proposed number		Number of questionnaires distributed	Number of responses	Proposed number
Clinical medicine and nursing	17	17(100%)	7(41.18%)		17	17(100%)	1(5.88%)
Education	11	9(81.82%)	9(81.82%)		9	9(100%)	2(22.22%)
Total	28	26(92.86%)	16(76.19%)		26	26(100%)	3(11.54%)

#### Experts’ authority and coordination

The expert authority coefficient (Cr) was the average value of expert familiarity with the indicators (Cs) and judgment criteria for the indicators. In this research, the expert familiarity was 0.946, judgement coefficient was 0.958, and the authority coefficient of the expert was 0.952, indicating that experts’ authority was relatively high and the result of the research was reliable and authoritative, as shown in Tables 3 and 4.

**Table 3:** Experts judgement

	High			Medium			Low	
	Numbers	Frequency(%)		Numbers	Frequency(%)		Numbers	Frequency(%)
Theoretical analysis	19	73.08		7	26.92		0	0
Practical experience	22	84.62		4	15.38		0	0
Reference	6	23.08		14	53.85		6	23.08
Intuitive perception	15	57.69		5	19.23		6	23.08

**Table 4:** Experts’ familiarity

Degree of familiarity	Very familiar		Relatively familiar		Familiar		Quite familiar		Completely not familiar	
	Numbers	Frequency	Numbers	Frequency	Numbers	Frequency	Numbers	Frequency	Numbers	Frequency
Self-assessment	19	73.08%	7	26.92%	0	0	0	0	0	0

In the first round of consultation, the Kendall’s W of course module and course contents were 0.291 and 0.321 respectively while in the second of consultation, they were 0.316 and 0.358 respectively. So the Kendall’s W test had statistical significance (*P*<0.05), indicating that the experts’ opinion coordination degree in the two rounds of consultation was preferable, as shown in Table 5.

**Table 5:** Experts’ coordination

Round	Index	Number of items	Kendall’s *W*	χ^2^	*P*
The first round	Course module	4	0.291	17.601	<0.001
	Course contents	33	0.321	189.823	<0.001
The second round	Competence module	5	0.316	21.017	<0.001
	Course contents	45	0.358	241.716	<0.001

#### Experts’ opinions on the training course

If the average score of the importance of the item was lower than 3.5 and the coefficient of variation was higher than 0.25, it meant that the item should be deleted. In the two rounds of expert consultation, the average score of the importance of the item was higher than 3.5 and the coefficient of variation was lower than 0.25, it indicated that the experts’ opinions were concentrated.

According to the opinions in the first round of the expert consultation, 1 course was deleted, 10 courses were added, 2 courses were advised and 1 course was split, while in the second round, only 2 courses were revised. Finally, a comprehensive description of the final content and methodology of the developed program was shown in Table 6, which was consist of 4 categories with 45 course contents and covered diverse teaching methods, such as face-to-face lectures, group discussion and reports, lectures, flipped class and computer-based operations. Among which the information awareness contained 7 courses, the information knowledge 15 courses, information ability 19 courses and information ethics 4 courses.

**Table 6:** Consultation result of training course of information literacy for clinical nurses (from the second round of the Delphi study)

Course module & course contents	Hours & teaching method	Average	Standard deviation	Variable coefficient	Weighting target
I Nursing information awareness		4.642	0.571	0.123	0.245
I-1Application of artificial intelligence and digitalization in nursing	2 h & lectures	4.501	0.512	0.114	0.022
I-2Overview of metacognitive theory	1 h & lecture	4.436	0.489	0.110	0.021
I-3Introduction to the information literacy for nursing staff in the context of big data	2 h & lectures	4.844	0.402	0.083	0.023
I-4Digital healthcare and digital nursing	2 h & lectures	4.603	0.463	0.101	0.022
I-5Application of information technology in nursing	2 h & flipped class	4.730	0.442	0.093	0.023
I-6Cultivation of information awareness of nursing staff	2 h & lectures	4.572	0.574	0.126	0.022
I-7Evidence-based nursing	2 h & lectures	4.435	0.584	0.132	0.021
II Nursing information knowledge		4.784	0.583	0.122	0.252
II-1Nursing knowledge on International Association for Health Information and Management Systems	1 h & lecture	4.438	0.573	0.129	0.021
II-2Values of nursing information	1 h & lecture	4.527	0.429	0.095	0.022
II-3Structure and function of the nursing information system	1 h & lecture	4.743	0.427	0.090	0.023
II-4Quality control knowledge of nursing informatization	2 h & lectures	4.459	0.525	0.118	0.021
II-5Standard of nursing informatization	2 h & lectures	4.643	0.538	0.116	0.022
II-6Nursing services on internet	2 h & group discussion	4.635	0.421	0.091	0.022
II-7Mobile nursing information system	2 h & lectures	4.589	0.438	0.095	0.022
II-8Maintenance of nurse workstation system	2 h & lectures	4.601	0.422	0.092	0.022
II-9Electronic medical record system & nursing paperwork system	1 h & lecture	4.662	0.416	0.089	0.022
II-10Usage specification of mobile medical PAD, carts, etc.	1 h & lecture	4.501	0.472	0.105	0.022
II-11Methodology of PDCA circle	2 h & group discussion	4.458	0.526	0.118	0.021
II-12Basic knowledge of information retrieval	2 h & lectures	4.607	0.465	0.101	0.022
II-13Computer network technology	2 h & computer practice	4.431	0.521	0.118	0.021
II-14Basic knowledge of wired and wireless network and network security knowledge	2 h & lectures	4.477	0.598	0.134	0.021
II-15Software of document management	2 h & computer practice	4.689	0.418	0.089	0.023
III Nursing information ability		4.841	0.325	0.067	0.255
III-1Collection of clinical nursing information and data	2 h & lectures	4.783	0.411	0.086	0.023
III-2Application of information technology in nursing work and healthcare education	1 h & lectures	4.799	0.407	0.085	0.023
III-3Operations of the nursing information system	1 h & computer practice	4.673	0.503	0.108	0.022
III-4Maintenance and management of nurse information system software and hardware	1 h & lecture	4.673	0.443	0.095	0.022
III-5The ability to collect nursing information	2 h & lectures	4.790	0.434	0.091	0.023
III-6The ability to identify the nursing information	2 h & lectures	4.771	0.473	0.099	0.023
III-7The ability to deal with nursing information	2 h & lectures	4.831	0.447	0.093	0.023
III-8The ability to manage nursing information	2 h & lectures	4.673	0.598	0.128	0.022
III-9Application of virtual information technology in nursing clinical education	2 h & lectures	4.504	0.463	0.103	0.022
III-10Retrieval and application of nursing literature	2 h & computer practice	4.612	0.433	0.094	0.022
III-11Retrieval and application of online resources	2 h & computer practice	4.638	0.495	0.107	0.022
III-12Promotion of scientific research on nursing based on big data	2 h & lectures	4.679	0.464	0.099	0.022
III-13Paper writing on informatization	2 h & lectures	4.682	0.475	0.101	0.022
III-14Statistical modeling and scientific research application of nursing data	2 h & lectures	4.710	0.457	0.097	0.023
III-15Nursing fund and project inspection and declaration	2 h & lectures	4.589	0.453	0.099	0.022
III-16Patent drafting and application	2 h & lectures	4.627	0.547	0.118	0.022
III-17Use of WPS Office application software and SPSS software	2 h & computer practice	4.782	0.429	0.090	0.023
III-18Making of multimedia courseware	2 h & computer practice	4.618	0.621	0.134	0.022
III-19Processing and editing of audios and images	2 h & computer practice	4.528	0.573	0.127	0.022
IV Nursing information ethics		4.695	0.488	0.104	0.248
IV-1Protection of software copyright, intellectual property and patent	1 h & lecture	4.615	0.420	0.091	0.022
IV-2Online code of ethics	1 h & lecture	4.682	0.325	0.069	0.022
IV-3Academic code of ethics	2 h & lectures	4.689	0.463	0.099	0.023
IV-4The right channels to obtain information resources	2 h & computer practice	4.783	0.437	0.091	0.023

### Application on the training course of information literacy for clinical nurses

Because hospitals in China have requirements for continuing education for clinical nurses, they organize nurses for continuing education every year, which can ensure that participants continuously participate in the research. Before the training course, we coordinate the time and location of the class with the hospital to ensure the implementation of the courses. The curriculum was implemented for the nurse from July to October 2022.

The general information of nurses in the two groups were shown in Table 7. The general demographic baseline data of the two groups were balance and comparable. Before the training, the difference in the information literacy of the nurses between the two groups was not statistically significant. After the training, the nurses in the observation group was significantly higher than that in the control group in terms of nursing information awareness, nursing information knowledge, nursing information ability and information ethics (*P*<0.05). Comparing the nurses in the same group before and after the training, the nurses in the observation group after the training was significantly higher than before the training in terms of nursing information awareness, nursing information knowledge, nursing information ability and information ethics (*P*<0.05), as shown in Table 8. This shows that the level of information literacy of nurses has been significantly improved after training especially in the aspect of information awareness, information knowledge, information ability and information ethics, and proves that information literacy training courses are scientific and effective.

**Table 7:** General information of clinical nurses in the two hospitals (n = 84)

Items	The control group(%)	The observation group(%)	χ^2^	*P*
Age				
<30	19(45.24)	20(47.62)	0.599	0.741
30–40	13(30.95)	10(23.81)		
>40	10(23.81)	12(28.57)		
Working experience				
<5	10(23.81)	12(28.57)	0.247	0.884
5–10	14(33.33)	13(30.95)		
>10	18(42.86)	17(40.48)		
Educational background				
College certificate	12(28.57)	14(33.33)	0.524	0.770
Bachelor degree	28(66.67)	25(59.52)		
Master degree and above	2(4.76)	3(7.14)		
Region				
Urban	30(71.43)	28(66.67)	0.223	0.637
Rural	12(28.57)	14(33.33)		
Marital status				
Unmarried	21(50.00)	18(42.86)	0.659	0.719
Married	20(47.62)	22(52.38)		
Widowed or divorced	1(2.38)	2(4.76)		
Professional title				
Nurse or nurse practitioner	15(35.71)	16(38.10)	0.720	0.698
Nurse in charge	23(54.76)	24(57.14)		
Associate chief nurse and above	4(9.52)	2(4.76)		
Post				
Head nurse	37(88.10)	38(90.48)	0.124	0.724
Nurse	5(11.90)	4(9.52)		
Whether or not attend the training courses				
Yes	37(88.10)	35(83.33)	0.389	0.533
No	5(11.90)	7(16.67)		

**Table 8:** Comparison of information literacy differences between the observation group and the control group

	Before the training		*t*	*P*	After the training		*t*	*P*
	The control group	The observation group			The control group	The observation group		
Information literacy	113.64 ± 7.59	114.05 ± 8.66	-0.580	0.564	112.74 ± 7.30	125.62 ± 8.36*	6.950	<0.01
Nursing information awareness	30.05 ± 3.84	29.12 ± 4.40	1.030	0.306	29.64 ± 3.63	32.76 ± 3.22*	4.262	<0.01
Nursing information knowledge	24.07 ± 3.10	24.14 ± 2.98	-0.108	0.914	23.98 ± 3.32	27.07 ± 2.87*	4.509	<0.01
Nursing information ability	15.38 ± 2.20	15.90 ± 3.96	-1.641	0.105	15.07 ± 2.04	17.81 ± 1.92*	7.590	<0.01
Information ethics	22.00 ± 2.55	22.24 ± 4.41	-0.303	0.762	22.00 ± 2.75	25.36 ± 2.44*	5.858	<0.01
Information support	22.14 ± 2.88	22.64 ± 3.64	0.136	0.487	22.05 ± 3.05	22.62 ± 3.37	0.766	0.448

Notes: * indicated the differences of the same group before and after the training (*P*<0.05)

## Discussion

Evidence-based practice is the integration of the best research evidence with clinical expertise and patient values. Evidence-based practice has been made possible by information technology. At present, with the rapid development of information technology and Evidence-Based Practice, nursing staff, as one of the largest groups in the medical and health field, must improve their information awareness and information ability [27, 28]. The level of information literacy of nursing staff was directly related to the quality of nursing services and directly linked to the quality of nursing service [29, 30]. Only by constantly mastering new knowledge and new technologies could nurses make full use of advanced information resources and meet the needs of the information-based medical society treatment [31]. Therefore, it is imperative to improve nurses’ information literacy in the background of the Evidence-based practice, and this is also the purpose and significance of our research.

Nowadays, the information curriculum for nursing staff was not valued in China, and the medical information curriculum was insufficient [32–34]. Based on this, our study constructed the training course of information literacy for nursing staff through a variety of methods such as literature review, group meetings, and Delphi expert consultation. Before the course contents were formulated, the research team held a group meeting to consult and listen to the opinions from clinical medical, nursing and education experts, and invited experts to make a feasibility assessment on the contents of the first draft of the course to ensure the practicability of the course contents. The finally information literacy training courses for clinical nurses constructed in our study included 4 course categories and 45 specific course contents. Among them, the nursing information awareness module contained 7 courses, the nursing information knowledge module 15 courses, the nursing information competence module 19 courses, and the nursing information ethics module 4 courses. The course contents were complete and comprehensive.

Among these course categories, Nursing information ability is given the largest proportion by experts, accounting for 0.255. When the nursing staff took their job in the hospital, in the case of many patients and few nursing staff in China, it was difficult for them to have time and energy to improve their professional quality through study [35, 36]. At the same time, they also face heavy work pressure [37, 38]. This shows that among nurses’ information literacy, their information ability is the most important and it is the basis of clinical nursing and to meet that demand nurses use all the skills of evidence-based practice. Nursing information knowledge is the second important course category in nurse information literacy training courses, which accounts for 0.252. Nursing information knowledge course category covers many knowledge of Nursing information, among which Software of document management knowledge is the most important, accounting for 0.023. Research indicates a significant role of new technologies in improving the effectiveness and efficacy of healthcare, and the knowledge of document management especially accurately document a patient’s status may literally mean life or death [39]. Evidence-based nursing practice in computer is a good approach to achieve this goal. Therefore, it is particularly important to comprehensively improve nursing information knowledge. Nursing information ethics is also a course category that cannot be ignored, which accounts for 0.248. In Nursing information ethics, the most important courses are academic code of technologies and the right channels to obtain information resources. Nursing information ethics is important during the nurse work. However, research shows that despite the importance and acceptance of research ethics consultation as an entity in many medical research areas, little is known about its status in nursing research [40]. Iran has taken nursing ethics, especially moral sensitivity, as an important part of nursing education, which provides a good reference for our nursing information ethics education [41]. We should promote nurses’ information ethics through the comprehensive improvement of nurses’ information literacy. Nursing information awareness is another course category of nursing information literacy training courses. Nursing staff are lack of information awareness and think it is the task of doctors to collect information in clinical work, as a result, they do not know how to make use of the electronic resources [42]. All these seriously hindered the progress and development of nursing staff. Nursing information awareness helps nurses to establish correct information awareness, which can be transformed into the search, acquisition and promotion of information knowledge [43]. In the category of Nursing information awareness courses, application of information technology in nursing and introduction to the information literacy for nursing staff are the most important, which both account for 0.023.

After constructing a systematic and comprehensive information literacy training course for nursing staff through two rounds of Delphi expert correspondence, we conducted an empirical study. From July to October 2022, a total of 84 clinical nurses from two hospitals were selected by the convenience sampling method, of which the nurses in one hospital were the control group and the nurses in the other hospital were the observation group. The general demographic data of the two groups were balanced, and there was no statistically significant difference in their information literacy level between the two groups of nurses before the training. 42 nurses in the observation group were trained by the constructed information literacy training course through face-to-face lectures, group discussion and reports, lectures, flipped class and computer-based operations. We compared the nursing staff’s information literacy level after training to verify the effectiveness of the training model. The results of the empirical study showed that the information literacy level of the nurses in the observation group after the training of the information literacy course was improved, and the scores in nursing information awareness, nursing information knowledge, nursing information ability, and information ethics were significantly higher than those in the control group after training (*P* < 0.05). There was no significant change in the scores of nursing staff in the two groups before and after the training in information support, which may be related to the fact that the hospital’s information support did not change in a short time. It could be popularized and applied around China to comprehensively improve the information literacy of nursing staff, improve the quality of nursing, provide reference for information training of clinical nurses, and promote the development of nursing disciplines [44, 45].

## Conclusion

The training course system covers Nursing information awareness, Nursing information knowledge, Nursing information ability and Nursing information ethics 4 course categories and 45 specific course contents. Besides, the effectiveness of the training course was verified through empirical research. Empirical research showed that information literacy training courses for clinical nurses could effectively improve their information literacy levels and could be further promoted and used.

### Limitations

Considering potential sources of bias or confounding factors, the extrapolation of the empirical study is limited, especially due to the use of convenience sampling method. Since the methods by which we evaluated nurses’ information literacy are subjective, in the future research, we will adopt a more objective method to measure the information literacy of nursing staff.

## Data Availability

The datasets generated during and analyzed during the current study are not publicly available due to the confidentiality announcement made on the participants but are available from the corresponding author upon reasonable request.
